# Valorization of Mango By-Products to Enhance the Nutritional Content of Maize Complementary Porridges

**DOI:** 10.3390/foods10071635

**Published:** 2021-07-15

**Authors:** Juliana Mandha, Habtu Shumoy, Athanasia O. Matemu, Katleen Raes

**Affiliations:** 1 Research Unit VEG-i-TEC, Department of Food Technology, Safety and Health, Ghent University Campus Kortrijk, Sint-Martens-Latemlaan 2B, 8500 Kortrijk, Belgium; Juliana.Mandha@UGent.be (J.M.); Habtu.Shumoy@UGent.be (H.S.); 2 Department of Food Biotechnology and Nutritional Sciences, Nelson Mandela African Institution of Science and Technology, Arusha 447, Tanzania; athanasia.matemu@nm-aist.ac.tz

**Keywords:** mango by-products, valorization, fortification, value addition, in vitro digestion, maize porridge

## Abstract

Mango by-products are disregarded as waste contributing to greenhouse gas emissions. This study used mango seed and kernel to enhance the nutritional content of maize complementary porridges. Composite maize-based porridges (MBP) were formulated by fortifying maize flour with fine ground mango seed and kernel at different levels (31%, 56%, 81%). The by-products and formulated porridges were characterized for their nutritional composition, mineral content, total phenolic content, and antioxidant capacity. Furthermore, the bioaccessibility of essential minerals during in vitro gastrointestinal digestion of the formulated porridges was determined using inductively coupled plasma optical emission spectrometry. Mango seed had a high fat (12.0 g/100 g dw) and protein content (4.94 g/100 g dw), which subsequently doubled the fat content of the porridges. Mango by-products increased the total phenolic content of maize porridge by more than 40 times and the antioxidant capacity by 500 times. However, fortification with mango by-products significantly decreased the bioaccessibility of minerals, especially manganese, copper, and iron, as the highest percentages of insoluble minerals were recorded in MBP 81 at 78.4%, 71.0%, and 62.1%, respectively. Thus, the results suggest that mango seed and kernel could increase the nutritional value of maize porridge, but fortification should be done at lower levels of about 31–56%.

## 1. Introduction

Fruit processing from industry and agriculture generates high amounts of by-products that are often disregarded as waste. This waste is decomposed in open fields or landfills and emits dangerous greenhouse gasses, such as methane, carbon dioxide, and nitrogen dioxide, which cause environmental problems [1]. Strategies to recycle of these fruit by-products back into the food system are essential to protect the environment and to reduce the social and economic burden.

Mango (*Mangifera indica* L.) is among the top 10 fruits of major economic importance cultivated in Africa. It belongs to the family of *Anacardiaceae* and is considered a high-quality fruit as the ‘King of fruits’ in the Orient [2]. Over 8 million tonnes of mangoes are produced in the region per year [3] and are often consumed either as fresh fruit or processed into various products such as pulp/juice, puree, pickle, jam, powder, and nectar. Mango processing generates approximately 3 million tonnes of the by-products (seeds, peels, kernels) making up 25–40% of fresh fruit. These are rich sources of nutrients and bioactive compounds, i.e., mango seed and kernel are excellent sources of dietary fiber, carotenoids, protein, fat, minerals, and phenolic compounds [4,5,6]. The bioactive compounds in mango by-products have antioxidant, antibacterial, cardioprotective, anti-inflammatory, and anti-proliferative properties [7] that protect against chronic non-communicable diseases. Hence, mango by-products could be transformed into useful products.

One possibility of utilizing mango by-products could be to use them to enrich the nutrient content of maize porridges through fortification. Maize (*Zea mays*) is a staple food that is largely consumed in developing countries (52 g/person/day) [8] and is the major complementary porridge for children. This food crop is mainly composed of carbohydrates (70%) and lacks essential micronutrients [9,10]. Food-to-food fortification is currently viewed as an emerging strategy against micronutrient deficiencies [11] and the use of locally available products is a cost-effective and sustainable strategy. Research on mango as a fortificant is limited and studies have mainly focused on the use mango pulp [12] but not its seed and kernel. Some authors have shown the use of mango pomaces to enrich wheat flour in the production of bakery products, such as cookies and biscuits [13,14] and starch-molded snacks [15], but not in maize porridges. Besides, investigations on how processes such as fortification and cooking alter the interactions of nutrients in the food matrix during in vitro gastrointestinal digestion broaden our understanding on how to utilize these by-products.

Therefore, the objectives of this study were to investigate the nutritional value, total phenolic content, antioxidant capacity, and minerals of mango seed, mango kernel, and maize flour, to formulate a composite maize porridge with the mango seed and kernel and to investigate the bioaccessibility of essential minerals in the porridges during in vitro gastrointestinal digestion.

## 2. Materials and Methods

### 2.1. Chemicals

ABTS (2,2′-azino-bis(3-ethylbenzothiazoline-6-sulfonic acid)), Trolox (6-hydroxyl-2,5,7,8-tetramethylchroman-2-carboxylic acid), DPPH (2,2-diphenyl-1-picrylhydrazyl), TFA (trifluoroacetic acid), gallic acid, Folin–Ciocalteu reagent (FC), pepsin from porcine gastric mucosa-lyophilized powder, α-amylase and pancreatin from porcine pancreas, heat-stable α-amylase solution, protease, amyloglucosidase solution, 2-(N-Morpholino) ethanesulfonic acid (MES), and Tris (hydroxymethyl) aminomethane (TRIS) were purchased from Sigma-Aldrich Co. (St. Louis, MO, USA). Technical grades of methanol (MeOH) (>98.5%), ethanol (C_2_H_5_OH) (93%), sodium hydroxide (NaOH) (97%), sodium chloride (NaCl) (97%), potassium persulfate (K_2_S_2_O_8_) (100%), sodium carbonate (Na_2_CO_3_) (>99%), nitric acid (HNO_3_) (>99%), sulfuric acid (H_2_SO_4_) (>97%), hydrochloric acid (HCl) (37.2% *w*/*w*), petroleum ether (C_6_H_14_) (30–60 °C, A.R), Kjeldahl tablet (CuH_10_O_9_S), potassium chloride (KCl) (> 99%), sodium hydrogen carbonate (NaHCO_3_) (>99%), sodium chloride (NaCl) (97%), magnesium chloride (MgCl_2_) (99%), ammonium chloride (NH_4_Cl) (100%), calcium chloride (CaCl_2_) (>93%), monopotassium phosphate (KH_2_PO_4_), and indicators (tashiro and phenolphthalein) were purchased from VWR International (Leuven, Belgium). Inductively coupled plasma (ICP) multi-element standard solution IV was procured from Merck KGak (Darmstadt, Germany).

### 2.2. Plant Materials

Mature mango fruits (*Mangifera indica, L*. cv Kagoogwa) and maize (*Zea mays*) flour were purchased from Nakasero market, Kampala, Uganda (latitude: 00°18′42.34″ N, longitude: 32°34′46.34′′ E). The mango fruits (10,000 g) were physically checked for integrity, insect contamination, and size/color uniformity. The screened samples were then packaged in air-tight boxes and cold transported by air to the Research Unit VEG-i-TEC of Ghent University, Kortrijk, Belgium. On arrival, the mango fruits were again inspected, washed, and the peel and fruit mesocarp was removed. The mango kernel was then manually separated from the mango seed and fine ground mango seed and kernel (wet basis) were obtained using a KitchenAid blender (Joseph, MI, USA). The obtained by-products were rapidly stored in sealed plastic bags at −20 °C until further analyses.

### 2.3. Sample Preparation 

**Figure 1:**
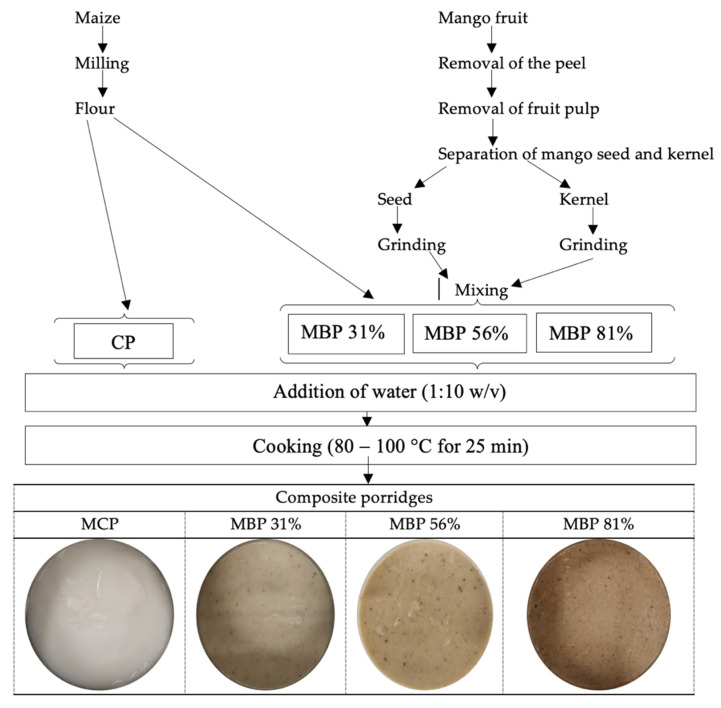
Flowchart showing the preparation of maize composite porridges (wt).

Using mass balance, all the porridge formulations were calculated to provide the recommended dietary intake (RDA) for energy (1200 kcal/day) at 300 g wt. Maize flour was substituted for fine ground mango seed and kernel at different levels—31%, 56%, and 81%—to formulate composite maize-blended porridges (MBP): MBP 31, MBP 56, and MBP 81, respectively. Porridge formulations are shown in Table 1. The maize control porridge (MCP) constituted of only maize flour and water (1:10 *m*/*v*). The flours were cooked into porridges using a traditional method, which involved cooking at 80–100 °C for 25 min (Figure 1).

### 2.4. Determination of Proximate Composition

The amounts of ash, fat, and soluble and insoluble dietary fiber of the raw materials (mango seed, kernel, maize flour) and composite porridges were assessed using AOAC standard methods, namely, 945.46, 920.39, and 991.43, respectively [16]. Moisture and protein concentrations were determined using ISO 1442-1973 and ISO 937-1978, respectively. A factor of 6.25 was used for conversion of nitrogen to crude protein. Digestible carbohydrates were calculated by difference, subtracting the sum of protein, ash, lipid, and total dietary fiber from 100.

### 2.5. Determination of Mineral Content 

The concentrations of essential minerals such as iron (Fe), zinc (Zn), manganese (Mn), copper (Cu), magnesium (Mg), potassium (K), calcium (Ca), and sodium (Na) were determined using the inductively coupled plasma optical emission spectrometry (ICP-OES) (Varian, PTY Ltd., Victoria, Australia) [17]. Briefly, 2 g of mango seed, mango kernel, and maize flour and 5 g of porridge sample were completely carbonized in a high-form porcelain crucible followed by overnight ashing in a muffle oven at 550 °C. The obtained ash was subsequently dissolved in 5 mL of 65% HNO_3_, filtered, and its mineral concentration measured using ICP-OES with Thermo iCAP 7200 spectrometer (Thermo Fisher Scientific Inc., Waltham, MA, USA). The ICP-OES was equipped with a peristaltic pump (0.76 mm), cyclonic spray chamber, concentric nebulizer, quartz plasma torch, and 2.0 mm alumina internal diameter injector. The instrumental parameters used were: 1180 W RF power, 12.0 L/min plasma flow rate, 0.5 L/min auxiliary gas flow rate, 0.5 L/min nebulizer flow rate, radial view, 15 min UV exposure time, 5 min VIS exposure time, 10 min warm up, and 40 min wash time. The wavelengths selected for Cu, Fe, Mn, Zn, K, Na, Ca, and Mg were 224.7, 239.6, 260.6, 202.6, 766.5, 589.6, 422.7, and 285.2 nm, respectively. Standard plot analytical curves for each element with a fit factor of above 0.99 were used to calculate the concentration of the elements in the samples in comparison to multi-element stock standard. Results were expressed as mg/100 g dry basis (dw) for the mango seed, kernel, maize flour, and mg/100 g wet basis (wt) for the porridges.

### 2.6. Determination of Total Phenolic Content 

Total phenolic compounds were extracted using 80% methanol as described by Gonzales et al. [18]. Briefly, either 2 g mango seed, 2 g mango kernel, 5 g maize flour, or 5 g porridge sample was added to 15 mL of methanol (MeOH 80%) and homogenized using Ultra Turrax homogenizer (T 18 digital, IKA, Staufen, Germany) at 1422× *g* (10,000 rpm) for 45 s and immediately kept on ice for 15 min. The homogenate was then centrifuged (Hermle Z300K, Hermle Labortechnik GmbH., Wehingen, Germany) at 2540× *g* (4000 rpm), 4 °C for 15 min and then filtered (filter paper particle retention 5–13 μm, VWR International., Leuven, Belgium) in the dark. The pellets were re-extracted with 10 mL MeOH (80%) using the same procedure. The collected supernatants were then pooled, filled to 25 mL with MeOH, and stored at −20 °C in the dark until further analyses.

Total phenolic content (TPC) was determined using the Folin–Ciocalteu (FC) method according to Huynh et al. [19] and Singleton et al. [20]. In brief, 1 mL methanolic extract was added to 1 mL deionized water and vortex (Vortex-genie 2, Thermo Fisher Scientific Inc., Waltham, MA, USA) mixed with 0.5 mL of 10 times diluted FC reagent in deionized water. After 6 min of standing, 1.5 mL Na_2_CO_3_ (20% *w*/*v*) and 1 mL deionized water were added, vortex mixed, and incubated in the dark for 2 h at room temperature. The absorbance of the mixture was then measured at 760 nm using a Spectrophotometer (Shimadzu UV-1800 spectrophotometer, Kioto, Japan) and the TPC concentration was expressed as mg gallic acid equivalent (GAE)/100 g of dried sample (dw).

### 2.7. Determination of Antioxidant Capacity

#### 2.7.1. Using ABTS Radical Scavenging Activity

Stock solution of ABTS^+^ was prepared by mixing equal amounts of 7 mM ABTS radical cation and 2.45 mM of potassium persulfate and allowing them to react for 12−16 h in the dark at room temperature. The working solution was subsequently prepared by diluting the stock solution with MeOH (90%) to an absorbance of 0.70 ± 0.02 at 734 nm equilibrated at 30 °C. Aliquots (20 μL) of each sample extract and standard Trolox solution or MeOH (90%) (blank) were then added to 2 mL of the ABTS^+^ solution, vortex mixed, and incubated for 5 min in the dark at room temperature. Thereafter, the absorbance of the resulting solution was measured spectrophotometrically (Shimadzu UV-1800 spectrophotometer, Kioto, Japan) at 734 nm, and results were expressed in mg Trolox equivalent (TE)/100 g of dried sample (dw) [21].

#### 2.7.2. Using DPPH Radical Scavenging Activity

The reducing ability of the antioxidants in the samples towards DPPH was measured using the procedure by Brand-Williams et al. [22]. Aliquots (200 μL) of sample extracts/Trolox standards solutions were vortex mixed for 10 s with 4 mL of DPPH solution (prepared by dissolving 3.94 mg DPPH in 100 mL pure MeOH) and incubated for 30 min at room temperature in the dark. The absorbance of the mixture was then measured using a spectrophotometer at 517 nm. Results were expressed in mg TE/100 g of dried sample (dw).

### 2.8. In Vitro Gastrointestinal Digestion

In vitro digestion of the fortified porridges was done using the international consensus static in vitro digestion method as described by Minekus et al. [23]. Porridge samples were digested in three simulated digestive solutions: salivary, gastric, and intestinal fluids simulating digestion in the mouth, stomach, and intestine, respectively. In the oral phase, 5 g of porridge was mixed thoroughly with 3.5 mL of simulated salivary fluid (KCl–15.1 mmol/L, KH_2_PO_4_–3.7 mmol/L, NaHCO_3_–13.6 mmol/L, MgCl_2_–0.15 mmol/L, (NH_4_)_2_CO_3_–0.06 mmol/L, pH 7), 0.5 mL α-amylase (0.15 g/mL–1500 units/mL), and 1000 µL 0.3 M CaCl_2_. The mixture was thoroughly homogenized, pH was adjusted to 7 using either 1 M NaOH or 1 M HCl, and then the mixture was incubated for 2 min at 37 °C with constant shaking in a warm water bath (Memmert WNB 45, Schwabach, Germany).

This was followed by the gastric phase, whereby the oral bolus was mixed with 7.5 mL of simulated gastric fluid (KCl–6.9 mmol/L, KH_2_PO_4_–0.9 mmol/L, NaHCO_3_–25 mmol/L, NaCl–47.2 mmol/L, MgCl_2_–0.1 mmol/L, (NH_4_)_2_CO_3_–0.5 mmol/L, pH 3), 1.6 mL of porcine pepsin (0.00781 g/mL–25,000 units/mL), and 700 µL 0.3 M CaCl_2_ made in simulated gastric fluid. The pH was adjusted to 3 using 1 M HCl. The mixture was then incubated at 37 °C in a shaking water bath for 90 min. Next, dialysis bags of 15.5 cm (molecular weight cut-off (MWCO), 12–14 kDa) containing 5.5 mL 0.9% NaCl and 5.5 mL 0.5 M NaHCO_3_ were inserted in the gastric chyme and incubation continued for further 30 min.

The gastric chyme was then thoroughly mixed with 11 mL of simulated intestinal fluid (KCl–6.8 mmol/L, KH_2_PO_4_–0.8 mmol/L, NaHCO_3_–85 mmol/L, NaCl–38.4 mmol/L MgCl_2_–0.33 mmol/L, pH 7), followed by 5.0 mL pancreatin (800 units/mL, 1 g in 250 mL), 2.5 mL of fresh bile prepared, 40 µL 0.3 M CaCl_2,_ 1.31 mL deionized water, and pH adjusted to 7 using either 1 M NaOH or 1 M HCl. The digestion continued at 37 °C for 2 h in a shaking water bath.

After the intestinal digestion phase, the dialysis bags were taken out, rinsed, and dried using a paper cloth and its contents (dialyzed minerals (D)) were transferred into falcon tubes. The remaining digestion solution was centrifuged at 2540× *g* (4000 rpm) for 15 min at 4 °C and supernatants had the soluble and non-dialyzable (SND) mineral while the pellets had insoluble minerals (P). The fractions were then oven dried, ashed, and solubilized using 1 M HNO_3_. Minerals were determined using inductively coupled plasma optical emission spectroscopy (ICP-OES) as previously described. The percentages of bioaccessible minerals were calculated using Equation (1).

D% = (D minerals/Total minerals (D + SND + P)) × 100(1)

SND% = (SND minerals/Total minerals (D + SND + P)) × 100 (2)

where D = dialyzed minerals, SND = soluble and non-dialyzable minerals, and P = insoluble minerals.

### 2.9. Determination of Porridge Viscosity

The porridge viscosity of each formulation was determined using a Brookfield DV2T viscometer (USA) with SNLV spindle at 37 °C, which is a suitable temperature considered for eating porridge. The viscometer was first standardized and the rotational speed ranging from 0.1 to 200 rpm was selected. Results with a torque between 10% and 100% were used and apparent viscosity measured as a function of shear rate (s^−1^) was presented in mPa·s.

### 2.10. Statistical Analysis

Data followed a normal distribution and variances were homogeneous. The experimental results were analyzed using one-way analysis of variance (ANOVA) using XL-STAT, (version 2020.1, Addinsoft, Paris, France), to check for any variations among treatments and comparison of means was done using multiple range test (Tukey’s HSD test). Significance difference was accepted at *p* < 0.05 and values are expressed as mean ± SD of two independent samples. Graphs were obtained using GraphPad Prism (Version 8.0.0 for macOS, San Diego, CA, USA). All the experiments were carried out in duplicates.

## 3. Results and Discussion

### 3.1. Nutrient Composition

Mango seed had the highest fat (12.0 g/100 g dw) and protein content (4.94 g/100 g dw) compared to mango kernel and maize flour (Table 2). The obtained protein values in the mango kernel were lower than the results by Mutua et al. [24] that showed an average of 6.74–9.20%. This could be attributed to differences fruit variety, geographical conditions, and climatic growing conditions. The ash in the mango seed was 1.39 g/100 g dw, similar to Bertha et al. [25] showing 1.29 g/100 g dw. Mango kernel had the highest insoluble (34.6 g/100 g dw) and soluble dietary fiber (5.07 g/100 g dw), while maize flour had the lowest total dietary fiber (4.82 g/100 g dw), although this was higher than prior reported results (2.68 g/100 g) [26]. Soluble fibers are known to reduce blood serum cholesterol, hence preventing the development of non-communicable diseases, and insoluble fibers improve regular bowel movements, hence reducing the risk of constipation and colon cancer by maintaining gut health [27]. Maize flour had a high carbohydrate of 81 g/100 g dw, which was consistent with previous analyses [9,10].

**Figure 2:**
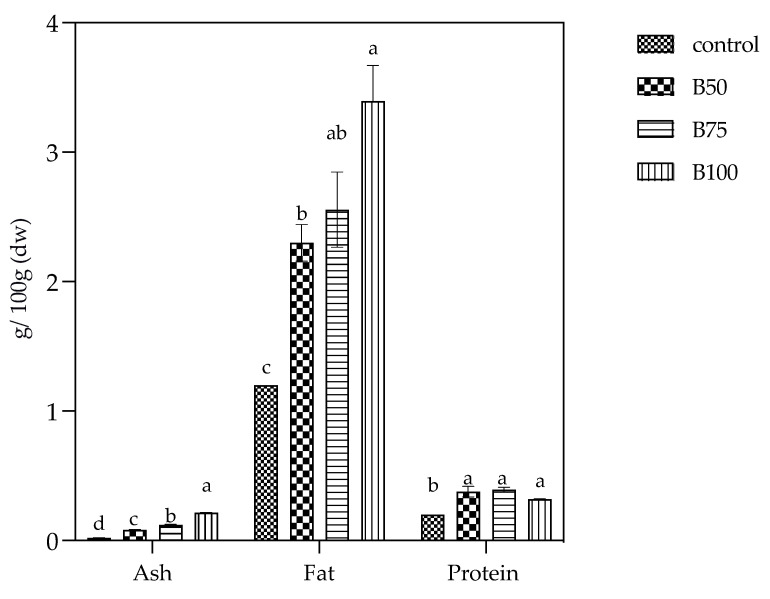
Ash, fat, and protein content of maize porridges. The results are expressed mean ± SD shown as error bars. Different lower-case letters (a–d) represent statistical differences. MCP is maize control porridge; MBP is formulated composite porridges.

After fortification of maize flour with mango seed and kernel, the composite maize porridges varied in their nutrient composition at the different levels of formulations as presented in Figure 2. Mango by-products significantly (*p* < 0.05) increased the protein, fat, and ash of the porridges, and this increment was proportional to the percentage of mango by-products. MBP 81 had the highest fat content at 3.29 g/100 g wt, as compared to MBP 31 at 2.30 g/100 g and the control 1.20 g/100 g (*p* = 0.001). Crude ash, which is an indicator of total minerals, ranged from 1.32 g/100 g wt in MBP 81 to 0.19 g/100 g wt in the MCP (*p* = 0.001), and the protein content was highest in MBP 31 and MBP 56 at 0.38 and 0.39 g/100 g (*p* = 0.004), respectively. Moisture content ranged from 83.6 in composite porridges to 89.4 g/100 g wt in the control. Maize starch granules are known to be insoluble in cold water but upon heating, they absorb water and swell due to the presence of amylose and amylopectin [28].

### 3.2. Mineral Content

Minerals are vital nutrients needed by the body to carry out different metabolic functions. The concentration of essential minerals in the mango seed and mango kernel were on average double that of maize flour (Table 3). Minerals such as Fe, Zn, Mn, K, Ca, and Mg are needed for the proper functioning of the body. Iron and Cu were highest in the mango kernel at 4.65 and 5.13 mg/100 g dw, respectively, and lowest in maize flour at 0.69 and 0.57 mg/100 g dw, respectively. Among the abundant elements, K and Ca had the highest values at 405 and 504 mg/100 g dw, respectively, in mango seed.

Table 4 shows the composition of trace (Cu, Fe, Mn, and Zn) and abundant (K, Na, Ca, and Mg) minerals in the porridges. Among the trace minerals, Fe, which plays an important role in the formation of hemoglobin, significantly differed (*p* = 0.030) in the porridges ranging from 0.36 to 0.49 mg/100 wt in the composite porridges compared to the control at 0.29 mg/100 g wt. Additionally, mango seed and kernel significantly (*p* < 0.05) increased the Mn and Zn amounts. Manganese is a scavenger of free radicals in the body and Zn improves the body’s immunity, especially in children. Regarding abundant minerals, Mg and Ca had the highest concentrations at 80.3 and 57 mg/100 g wt, respectively, and their amounts did not vary with the fortification of mango fruit by-products. However, there was a significant (*p* = 0.001) increment of K from 7.02 mg/100 g wt in the control to 40.7 mg/100 g wt in MBP 81 porridge.

### 3.3. Total Phenolic Content and Antioxidant Capacity

Phenolic compounds are a major group of phytochemicals commonly distributed in plants. Mango seed was observed as a potentially good source of total phenolic compounds as it had the highest TPC of 3714 mg GAE/100 g dw, followed by mango kernel at 263 mg GAE/100 g dw (Table 5). These results are comparable with previous findings of Bertha el al. [25] and Nguyen el al. [29] wherein the TPC of tropical mango seed was 8.95–12.8 g/100 g dw. The distribution of total phenolic compounds could be affected by the type of crop, as maize flour had the lowest TPC of 18.7 mg GAE/100 g dw. These compounds have anti-inflammatory, antioxidant, and anti-proliferative properties protecting the human body from infections. The antioxidant activity was measured by two different methods: ABTS^+^ and DPPH. The mango seed had the highest antioxidant activity according to DPPH at 10,659 mg TE/100 g dw and ABTS^+^ at 10,568 mg TE/100 g dw.

Interestingly, mango by-products increased the TPC of maize porridge by more than 40 times (Table 5). The results showed that higher fortification of maize flour with mango by-products (seed and kernel) resulted in higher TPC values of (*p* < 0.05) from 2.2 mg GAE/100 g wt in MCP to 479 mg GAE/100 g wt in MBP 81. Bertha et al. [25] reported the main antioxidant polyphenol compounds in mango seed as gallic acid > ellagic acid > mangiferin > catechin > gallocatechin > gallocatechin gallate. These compounds could be responsible for the TPC of the porridges. These health-promoting substances are essential in the removal of free radicals as antioxidant agents. Similarly, the antioxidant capacity of the porridges using both ABTS^+^ and DPPH increased with the fortification of mango by-products. The antioxidant capacity could also be attributed to other compounds besides phenolic compounds in the porridges such as vitamin C and carotenoids, among others. However, since the porridges are cooked at high temperatures (>80 °C), it can be assumed that vitamin C is degraded, and does not play a role in the antioxidant capacity of the porridges.

### 3.4. Bioaccessibility of Minerals during In Vitro Gastrointestinal Digestion

**Figure 3:**
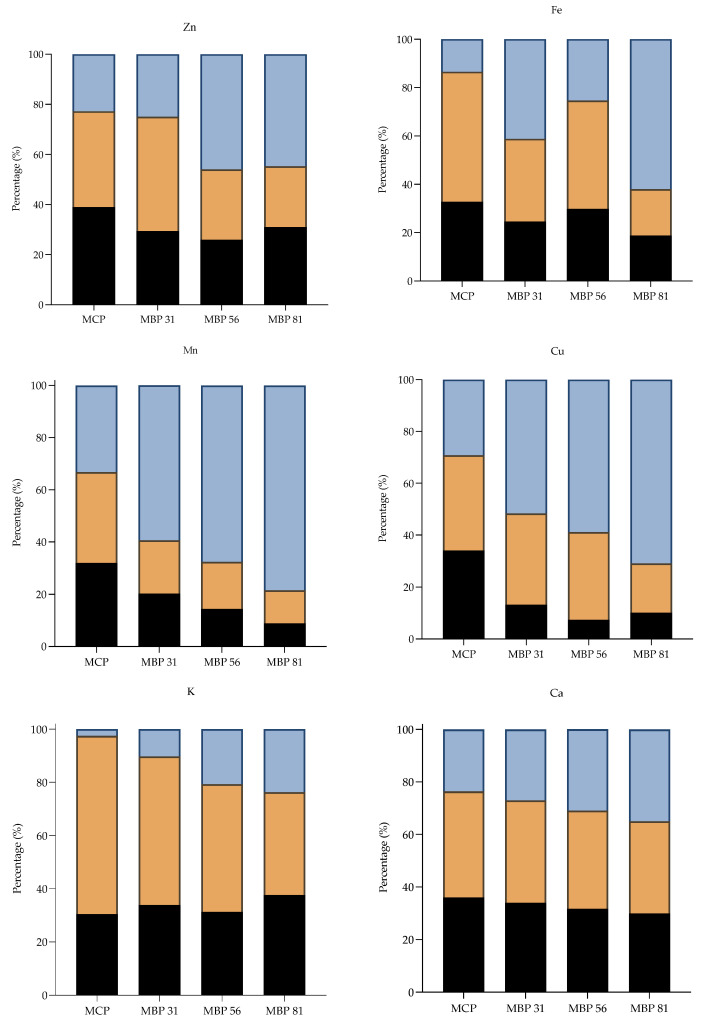

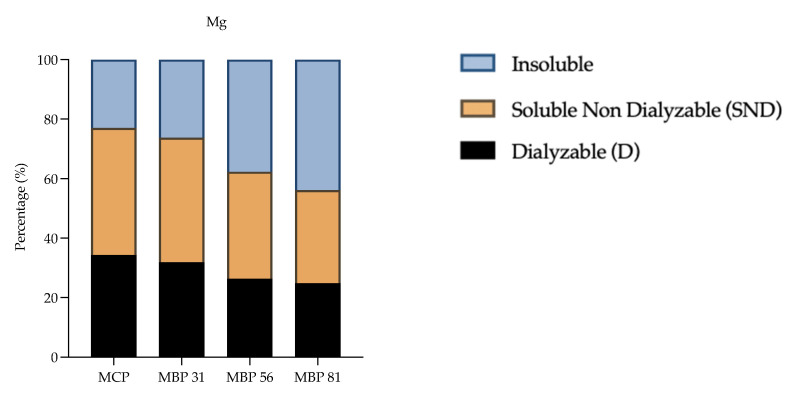
Percentage (%) bioaccessibility of dialyzable (D), soluble non-dialyzable (SND), and insoluble minerals during in vitro digestion in the formulated composite porridges.

Mineral content was investigated during in vitro gastrointestinal digestion, which predicts the proportion of nutrients released from food along the gastrointestinal tract making the nutrients available for absorption into the bloodstream for biological functions [23]. Overall, the percentage of bioaccessible, i.e., dialyzable and soluble non-dialyzable minerals, were significantly (*p* < 0.05) higher in MCP as compared to the MBP composite porridges (Figure 3). Among the essential micro minerals, zinc had the highest dialyzable percentage (38.9%), followed by Cu (34.0%) and Fe (32.7%). Fortification with mango by-products significantly (*p* < 0.05) decreased the bioaccessibility of micro minerals, especially Mn, Cu, and Fe, as the highest percentages of insoluble minerals were recorded in MBP 81 in at 78.4%, 71.0%, and 62.1%, respectively. Similarly, there was a gradual decrease in SND% and D% of macro minerals such as K and Mg (*p* < 0.05) in correlation with an increased proportion of fortification with mango seed and kernel.

The observed lower proportions of bioaccessible minerals in the composite porridges than the control may be likely due to the presence of considerable amounts of phenolic compounds, phytates, and tannins in the mango by-products [30] that could have a pronounced effect of binding minerals, thus forming complexes that are insoluble for absorption. Additionally, as previously shown in Table 2, the mango seed and mango kernel had higher amounts of soluble and insoluble dietary fibers than maize flour, which could consequently influence the bioaccessibility of minerals. Dietary fibers decrease the diffusion kinetics of mineral sources by binding and physically entrapping minerals [31,32]. Fernández et al. [33] suggested that bioaccessibility of minerals could be affected by interaction with other components in the digested food. Minerals may also interfere with each other’s absorption [34], for example; the presence of Ca impairs Zn absorption due to co-precipitation, Mn affects Fe absorption as the intestine cannot differentiate them, and finally, Zn and Fe compete for intestinal absorption due to similar configuration [35,36].

### 3.5. Viscosity of the Porridges

**Figure 4:**
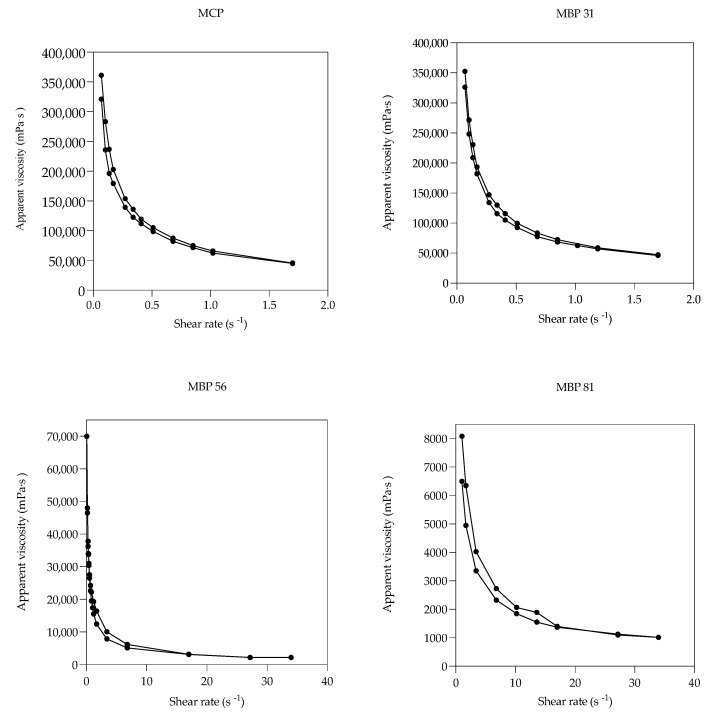
Effect of mango by-products on the apparent viscosity (mPa·s) of maize-based porridges expressed as a factor of shear rate (s^−1^).

From the data in Figure 4, it can be seen that the apparent viscosity of all the porridges generally decreased with increasing shear rate; hence, it followed non-Newtonian characteristics of pseudoplastic fluids. However, this change was observed to be reversible as the decreasing shear rate had similar apparent viscosity with increasing shear rate. These porridges therefore have thixotropic properties as they revert to their original state on standing [37]. Viscosity is an important quality parameter for the utilization of starch-based foods and is desirable when adequate solid content is maintained and allows for ease of consumption.

It was further observed that at a constant initial shear rate of 0.068 s^−1^, viscosity differed significantly (*p* < 0.05) across the porridges. The control porridge and MBP 31 had the highest viscosities at 321,300 and 352,500 mPa·s, respectively, unlike MBP 56 and MBP 81, which registered lower viscosities of 70,000 and 15,000 mPa·s, respectively. A highly dense porridge reduces food/energy intake and may not be easily palatable by children. The high viscosities of MCP and MBP 31 porridges could be due to their greater proportion of starch, as starch granules gelatinize during cooking, increasing viscosity. Additionally, soluble fibers in the mango seed and kernel may absorb and retain moisture, increasing viscosity and gel formation [38]. However, fortification of maize flour with mango by-products (seed and kernel) at higher levels (>50%) significantly decreased (*p* < 0.05) porridge viscosity, possibly due to the insoluble solids and fat in these porridges (Table 2), as fat forms insoluble complexes with starch granules and/or forms a fatty layer around starch granules, reducing their water absorption capacity during cooking, and hence reducing viscosity [39]. A porridge with low viscosity but with high energy density is desirable for infants and young children for easy mastication and swallowing.

## 4. Conclusions

This study was conducted to enhance the nutritional content of maize complementary porridges with mango by-products (seed and kernel). Based on the results, mango seed and kernel increased the protein, fat, and mineral (Fe, Mn, Zn) content of maize porridges in proportion to the level of fortification. In addition, the by-products increased the total phenolic content of maize porridges, which subsequently improved their antioxidant capacity. However, the by-products decreased mineral bioaccessibility during in vitro gastrointestinal digestion. Therefore, fortification using mango seed and kernel should be done at lower levels of about 31–56%. Further studies to understand the structures of the mineral inhibitors in mango seed and kernel may be conducted. Overall, this work provides an alternative way of utilizing agro-industrial by-products in the formulation of food products.

## Figures and Tables

**Table 1:** Formulations of the composite porridges (g per 300 g wt).

Raw Materials	Composite Porridge Formulation			
	MCP	MBP 31%	MBP 56%	MBP 81%
Maize flour (g)	300	206.2	131.9	57.6
Mango seed (g)	0	66.1	129.3	192.5
Mango kernel (g)	0	27.7	38.8	49.9
Energy (kcal)	1213.7	1200	1200	1200

**Table 2:** Nutrient composition (g/100 g dw) of mango by-products and maize flour. The results are expressed as mean ± SD.

	Mango Kernel	Mango Seed	Maize Flour
Moisture	36.6 ± 0.02	51.7 ± 0.01	6.14 ± 0.04
Carbohydrate	18.5 ± 7.82	6.85 ± 3.15	81.26 ± 0.95
Fat	2.92 ± 0.02	12.0 ± 1.43	4.26 ± 0.63
Protein	1.58 ± 0.07	4.94 ± 0.09	3.29 ± 0.14
Ash	0.70 ± 0.07	1.39 ± 0.06	0.23 ± 0.05
Soluble dietary fiber	5.07 ± 0.76	1.83 ± 0.93	2.21 ± 0.06
Insoluble dietary fiber	34.6 ± 7.04	21.6 ± 7.41	2.61 ± 0.06
Total dietary fiber	39.7 ± 7.80	23.4 ± 8.45	4.82 ± 0.00

**Table 3:** Mineral composition of mango by-products and maize flour (mg/100 g dw). The results are expressed as mean ± SD.

Mineral	Mango Kernel	Mango Seed	Maize Flour
Copper, Cu	5.13 ± 0.27	3.39 ± 0.51	0.57 ± 0.04
Iron, Fe	4.65 ± 0.16	4.29 ± 0.23	0.69 ± 0.06
Manganese, Mn	1.14 ± 0.07	1.42 ± 0.03	0.18 ± 0.04
Zinc, Zn	2.01 ± 0.08	2.01 ± 0.43	0.74 ± 0.09
Potassium, K	163 ± 19	405 ± 28	76.7 ± 4.2
Sodium, Na	103 ± 6	139 ± 10	29.6 ± 0.5
Calcium, Ca	395 ± 24	504 ± 14	82.8 ± 0.4
Magnesium, Mg	76.5 ± 2.8	121 ± 1	19.5 ± 0.2

**Table 4:** Mineral composition of formulated maize porridges (mg/100 g wt). The results are expressed as mean ± SD. Different lower-case letters represent statistical differences.

Mineral	MCP	MBP 31	MBP 56	MBP 81	*p* Value
Copper, Cu	0.32 ± 0.02 ^a^	0.39 ± 0.04 ^a^	0.37 ± 0.03 ^a^	0.34 ± 0.02 ^a^	0.349
Iron, Fe	0.29 ± 0.04 ^b^	0.36 ± 0.02 ^ab^	0.49 ± 0.03 ^a^	0.47 ± 0.03 ^ab^	0.030
Manganese, Mn	0.08 ± 0.01 ^c^	0.12 ± 0.01 ^bc^	0.15 ± 0.01 ^b^	0.22 ± 0.01 ^a^	0.001
Zinc, Zn	0.38 ± 0.02 ^b^	0.28 ± 0.00 ^b^	0.47 ± 0.00 ^ab^	1.06 ± 0.21 ^a^	0.021
Potassium, K	7.02 ± 0.53 ^c^	16.3 ± 0.6 ^bc^	25.36 ± 0.93 ^b^	40.7 ± 3.7 ^a^	0.001
Sodium, Na	22.8 ± 2.5 ^a^	24.8 ± 2.6 ^a^	19.9 ± 0.2 ^a^	23.2 ± 1.3 ^a^	0.432
Calcium, Ca	57.4 ± 2.5 ^a^	55.1 ± 7.1 ^a^	57.0 ± 0.5 ^a^	54.8 ± 3.1 ^a^	0.956
Magnesium, Mg	63.3 ± 3.8 ^a^	68.7 ± 8.3 ^a^	71.7 ± 0.1 ^a^	80.3 ± 4.0 ^a^	0.256

**Table 5:** Total phenolic content and antioxidant capacity of mango by-products, maize flour, and formulated composite porridges. The results are expressed as mean ± SD. Different lower-case letters represent statistical differences.

	TPC (GAE)	Antioxidant Capacity	
		ABTS (TE)	DPPH (TE)
Materials (mg/100 g dw)			
Mango kernel	263 ± 0.59	745 ± 13.45	670 ± 2.93
Mango seed	3714 ± 11.91	10568 ± 73.05	10659 ± 419.69
Maize flour	18.7 ± 0.00	15.66 ± 5.49	4.08 ± 1.30
Formulated Porridges (mg/100 g wt)			
MCP	2.20 ± 0.09 ^d^	12.5 ± 0.90 ^d^	3.46 ± 0.09 ^d^
MBP 31	131 ± 3.46 ^c^	853 ± 42.5 ^c^	530 ± 1.86 ^c^
MBP 56	309 ± 1.87 ^b^	1569 ± 80.3 ^b^	1245 ± 18.0 ^b^
MBP 81	479 ± 11.08 ^a^	3846 ± 22.7 ^a^	2276 ± 17.9 ^a^
*p*-value	0.000	0.000	0.000

## Data Availability

The authors confirm that the data supporting the findings of this study are available within the article.

## References

[1] Sagar N.A., Pareek S., Sharma S., Yahia E.M., Lobo M.G. (2018). Fruit and vegetable waste: Bioactive compounds, their extraction, and possible utilization. Compr. Rev. Food Sci. Food Saf..

[2] Ahmed J., Ramaswamy H.S., Hiremath N. (2005). The effect of high pressure treatment on rheological characteristics and colour of mango pulp. Int. J. Food Sci. Technol..

[3] FAOSTAT (2020). Food and Agriculture Data. http://www.fao.org/faostat/en/#data/QC.

[4] Muchiri D.R., Mahungu S.R., Gituanja S.N. (2012). Studies on mango (*Mangifera indica* L.) kernel fat of some Kenyan varieties in Meru. J. Am. Oil. Chem. Soc..

[5] Singh D., Siddiq M., Greiby I., Dolan K.D. (2013). Total phenolics, antioxidant activity, and functional properties of ‘Tommy Atkins’ mango peel and kernel as affected by drying methods. Food Chem..

[6] Jahurul M.H.A., Zaidul I.S.M., Ghafoor K., Al-Juhaimi F.Y., Nyam K.L., Norulaini N.A., Sahena F., Omar A.K.M. (2015). Mango (*Mangifera indica* L.) by-products and their valuable components: A review. Food Chem..

[7] Castro-vargas H.I., Vivas D.B., Barbosa J.O., Johanna S., Medina M., Aristizabal F., Parada-alfonso F. (2019). Bioactive phenolic compounds from the agroindustrial waste of Colombian mango cultivars ‘Sugar Mango’ and ‘Tommy Atkins’—An alternative for their use and valorization. Antioxidants.

[8] Ranum P., Peña-Rosas J.P., Garcia-Casal M.N. (2014). Global maize production, utilization, and consumption. Ann. N. Y. Acad. Sci..

[9] Qamar S., Aslam M., Huyop F., Javed M.A. (2017). Comparative study for the determination of nutritional composition in commercial and noncommercial maize flours. Pak. J. Bot..

[10] Shiriki D., Igyor M.A., Gernah D.I. (2015). Nutritional evaluation of complementary food formulations from maize, soybean and peanut fortified with moringa oleifera leaf powder. Food Nutr. Sci..

[11] Kruger J., Taylor J.R.N., Ferruzzi M.G., Debelo H. (2020). What is food-to-food fortification? A working definition and framework for evaluation of efficiency and implementation of best practices. Compr. Rev. Food Sci. Food Saf..

[12] Pobee R.A., Johnson P.N.T., Akonor P.T., Buckman S.E. (2017). Nutritional, pasting and sensory properties of a weaning food from rice (*Oryza sativa*), soybeans (*Glycine max*) and kent mango (*Mangifera indica*) flour blends. Afr. J. Food Agric. Nutr. Dev..

[13] Ashoush I.S., Gadallah M.G.E. (2011). Utilization of mango peels and seed kernels powders as sources of phytochemicals in biscuit. World J. Dairy Food Sci..

[14] Awolu O.O. (2018). Influence of defatted mango kernel seed flour addition on the rheological characteristics and cookie making quality of wheat flour. Food Sci. Nutr..

[15] Blancas-benitez F.J., Avena-bustillos R.D.J., Montalvo-gonzález E., Sáyago-ayerdi S.G., Mchugh T.H. (2015). Addition of dried ‘Ataulfo’ mango (*Mangifera indica* L.) by-products as a source of dietary fiber and polyphenols in starch molded mango snacks. J. Food Sci. Technol..

[16] AOAC (2010). Official Methods of Analysis of Association of Official Analytical Chemists (AOAC). Official Methods of Analysis of AOAC International.

[17] Ashoka S., Peake B.M., Bremner G., Hageman K.J., Reid M.R. (2009). Comparison of digestion methods for ICP-MS determination of trace elements in fish tissues. Anal. Chim. Acta.

[18] Gonzales G.B., Smagghe G., Raes K., Van camp J. (2014). Combined alkaline hydrolysis and ultrasound-assisted extraction for the release of nonextractable phenolics from cauli flower (*Brassica oleracea var. botrytis*) waste. J. Agric. Food Chem..

[19] Huynh N.T., Smagghe G., Gonzales G.B., Van camp J., Raes K. (2014). Enzyme-assisted extraction enhancing the phenolic release from cauliflower (*Brassica oleracea L. var. botrytis)* outer leaves. J. Agric. Food Chem..

[20] Singleton V.L., Orthofer R., Lamuela-Raventós R.M. (1999). Analysis of total phenols and other oxidation substrates and antioxidants by means of folin-ciocalteu reagent. Methods Enzymol..

[21] Re R., Pellegrini N., Proteggente A., Pannala A., Yang M., Rice-Evans C. (1999). Antioxidant activity applying an improved Abts radical cation decolorization assay. Free Radic. Biol. Med..

[22] Brand-Williams W., Cuvelier M.E., Berset C. (1995). Use of a free radical method to evaluate antioxidant activity. LWT–Food Sci. Technol..

[23] Minekus M., Alminger M., Alvito P., Ballance S., Bohn T., Bourlieu C., Carrière F., Boutrou R., Corredig M., Dupont D. (2014). Standardised static in vitro digestion method suitable for food-an international consensus. Food Funct..

[24] Mutua J.K., Imathiu S., Owino W. (2017). Evaluation of the proximate composition, antioxidant potential, and antimicrobial activity of mango seed kernel extracts. Food Sci. Nutr..

[25] Bertha C.T., Alberto S.B.J., Tovar J., Sáyago-Ayerdi S.G., Zamora-Gasga V.M. (2019). In vitro gastrointestinal digestion of mango by-product snacks: Potential absorption of polyphenols and antioxidant capacity. Int. J. Food Sci. Technol..

[26] Rai S., Kaur A., Singh B. (2014). Quality characteristics of gluten free cookies prepared from different flour combinations. J. Food Sci. Technol..

[27] Celia M., Hauly D.O. (2002). Inulin and oligofructosis: A review about functional properties, prebiotic effects and importance for food industry. Semin. Ciênc. Exatas Tecnol..

[28] Kaur H., Gill B.S., Karwasra B.L. (2018). In vitro digestibility, pasting, and structural properties of starches from different cereals. Int. J. Food Prop..

[29] Nguyen N.M.P., Le T.T., Vissenaekens H., Gonzales G.B., Van Camp J., Smagghe G., Raes K. (2019). In vitro antioxidant activity and phenolic profiles of tropical fruit by-products. Int. J. Food Sci. Technol..

[30] Abdalla A.E.M., Darwish S.M., Ayad E.H.E., El-Hamahmy R.M. (2007). Egyptian mango by-product 1. Compositional quality of mango seed kernel. Food Chem..

[31] Baye K., Guyot J.P., Mouquet-Rivier C. (2017). The unresolved role of dietary fibers on mineral absorption. Crit. Rev. Food Sci. Nutr..

[32] Sanz-Penella J.M., Laparra J.M., Sanz Y., Haros M. (2012). Bread supplemented with amaranth (*Amaranthus cruentus*): Effect of phytates on in vitro iron absorption. Plant. Foods Hum. Nutr..

[33] Fernández-García E., Carvajal-Lérida I., Pérez-Gálvez A. (2009). In vitro bioaccessibility assessment as a prediction tool of nutritional efficiency. Nutr. Res..

[34] Gibson R.S. (1994). Content and bioavailability of trace elements in vegetarian diets. Am. J. Clin. Nutr..

[35] Hemalatha S., Platel K., Srinivasan K. (2007). Zinc and iron contents and their bioaccessibility in cereals and pulses consumed in India. Food Chem..

[36] Sandstrom B. (2001). Micronutrient interactions: Effects on absorption and bioavailability. Br. J. Nutr..

[37] Bourne M.C. (2002). Food Texture and Viscosity: Concept and Measurement.

[38] Corradini C., Lantano C., Cavazza A. (2013). Innovative analytical tools to characterize prebiotic carbohydrates of functional food interest. Anal. Bioanal. Chem..

[39] Arocha M., De E., Gómez M., Rosell C.M. (2012). Effect of different fibers on batter and gluten-free layer cake properties. LWT–Food Sci. Technol..

